# Selection signatures of Fuzhong Buffalo based on whole-genome sequences

**DOI:** 10.1186/s12864-020-07095-8

**Published:** 2020-09-29

**Authors:** Ting Sun, Guang-yun Huang, Zi-hao Wang, Shao-hua Teng, Yan-hong Cao, Jun-li Sun, Quratulain Hanif, Ning-bo Chen, Chu-zhao Lei, Yu-ying Liao

**Affiliations:** 1 Animal Husbandry Institute of Guangxi Zhuang Autonomous Region, Guangxi Key Laboratory of Livestock Genetic Improvement, Nanning, 530001 China; 2 College of Animal Science and Technology, Northwest A&F University, Yangling, 712100 Shaanxi China; 3 Computational Biology Laboratory, Agricultural Biotechnology Division, National Institute for Biotechnology and Genetic Engineering, Faisalabad, Pakistan; 4 Department of Biotechnology, Pakistan Institute of Engineering and Applied Sciences, Nilore, Islamabad, Pakistan

**Keywords:** Buffalo, Resequencing, Selection signatures

## Abstract

**Background:**

Fuzhong buffalo, a native breed of Guangxi Zhuang Autonomous Region, is traditionally used as a draft animal to provide farm power in the rice cultivation. In addition, the Fuzhong buffalo also prepared for the bullfighting festival organized by the locals. The detection of the selective signatures in its genome can help in elucidating the selection mechanisms in its stamina and muscle development of a draft animal.

**Results:**

In this study, we analyzed 27 whole genomes of buffalo (including 15 Fuzhong buffalo genomes and 12 published buffalo genomes from Upper Yangtze region). The ZHp, ZFst, π-Ratio, and XP-EHH statistics were used to identify the candidate signatures of positive selection in Fuzhong buffalo. Our results detected a set of candidate genes involving in the pathways and GO terms associated with the response to exercise (e.g., *ALDOA*, *STAT3*, *AKT2*, *EIF4E2*, *CACNA2D2*, *TCF4*, *CDH2*), immunity (e.g., *PTPN22*, *NKX2-3*, *PIK3R1*, *ITK*, *TMEM173*), nervous system (e.g., *PTPN21*, *ROBO1*, *HOMER1*, *MAGI2*, *SLC1A3*, *NRG3*, *SNAP47*, *CTNNA2*, *ADGRL3*). In addition, we also identified several genes related to production and growth traits (e.g., *PHLPP1*, *PRKN*, *MACF1*, *UCN3, RALGAPA1*, *PHKB*, *PKD1L*). Our results depicted several pathways, GO terms, and candidate genes to be associated with response to exercise, immunity, nervous system, and growth traits.

**Conclusions:**

The selective sweep analysis of the Fuzhong buffalo demonstrated positive selection pressure on potential target genes involved in behavior, immunity, and growth traits, etc. Our findings provided a valuable resource for future research on buffalo breeding and an insight into the mechanisms of artificial selection.

## Background

Buffalo has been an important livestock animal used as a source of food and draught power in tropical and subtropical regions. It can be divided into two types: river (2*n =* 50) and swamp buffalo (2*n =* 48) [1]. China is one of the countries with the largest population of swamp buffalo. A recent study showed that the swamp buffalo can be divided into two divergent groups: Southeast Asian buffalo (including buffaloes from Southeast Asia and Southwest China) and South China buffalo (including the buffalo from Upper Yangtze and Middle-Lower Yangtze) [2]. In particular, the buffalo distributed in Upper Yangtze exhibited weak gene flow from the Southeast Asian buffalo [2]. The Fuzhong (FZ) buffalo is a native livestock from Guangxi Zhuang Autonomous Region, one of the hottest and most humid region in the Southwest of China [3]. Except for its use as a draft animal like other swamp buffaloes, the FZ buffalo has also been used in bullfighting as a folk custom with long history in Guangxi Zhuang Autonomous Region [4]. The FZ buffaloes have been adaptive to the local climate with enhanced disease resistance and heat tolerance. To date, its genomic value and potential are yet to be discovered.

Recent developments of high-throughput sequencing and genotyping technologies allow the construction of detailed selection signature maps for human and various domestic animals. With the growing importance of buffalo industry, the sequencing and genotyping platforms have been used to investigate the genetic diversity, demographic history, and selective signatures, etc. Recently, a 90 K SNPChip for buffalo (Axiom® Buffalo 90 K Genotyping Array) has been developed, which includes about 90 K SNP loci covering the river buffalo genome [5]. Up to now, most studies mainly focused on the dairy buffalo. A previous investigation revealed clear divergence of two native Iranian buffalo breeds (Azeri and Khuzestani) and pointed to candidate genes for milk production, growth, immunity and nervous system, etc. [6]. Another study detected genes associated with the body size, immunity and coat color by analyzing the ROH of the Azeri and Khuzestani buffalo breeds using the same dataset [7]. The same chip has also been used for the GWAS analysis of water buffalo, revealing a set of candidate genes associated with milk traits (e.g., *MFSD14A*, *SLC35A3*, *PALMD*, *RGS22*) [8]. Furthermore, there were few studies performed at the whole genome level. Whitacre et al. detected 13 genes potentially involved in the development of the hindlimbs based on the whole genome sequence of buffalo [9]. A recent study described the genetic history and population structure of swamp and river buffalo by analyzing 121 whole genomes from 25 breeds with different geographical origins [2], intercepting the candidate genes associated with nervous system and muscle development in swamp buffalo, while genes related to heat-stress and immunity were detected in the river buffalo [2].

To date, several methods are employed to detect the selective sweeps in various livestock genomes. In the current study, the fixation index (Fst) was used to measure the genetic differentiation between populations [10]. The larger Fst value indicates the difference in the two populations. π-Ratio was used to identify the differences in nucleotide divergence between populations. Rubin et al. defined and applied a Z-score test for heterozygosity depression (ZHp) on chicken genome sequence, which basically expresses how much the expected heterozygosity in chromosome windows deviate from the average genome heterozygosity [11]. The extremely low ZHp scores indicate putative selective sweeps because of excess homozygosity. Moreover, the cross-population extended haplotype homozygosity test (XP-EHH) is used to detect ongoing or nearly fixed selective sweeps by comparing haplotypes between the two populations [12].

In the present study, 27 whole genomes of buffalo (including 15 newly sequenced FZ buffalo genomes and 12 published buffalo genomes from regions of Upper Yangtze (UY) were analyzed. The above methods (Fst, ZHp, π-Ratio, and XP-EHH) were used to search for the candidate signatures of positive selection for FZ buffalo.

## Results

### Genome resequencing of Fuzhong buffalo

**Fig. 1:**
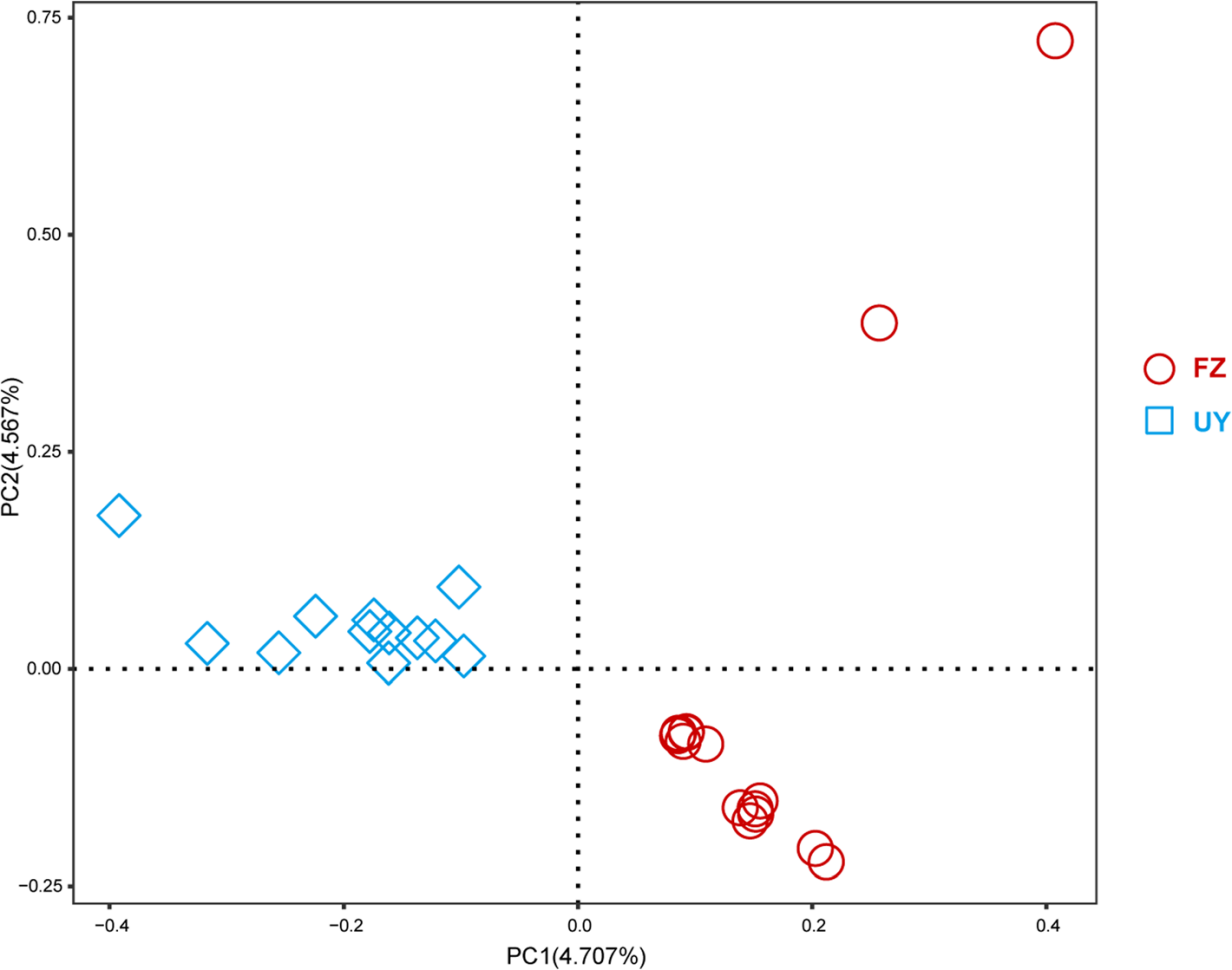
Principal component analyses (PCA) of the FZ and UY buffaloes. FZ: Fuzhong buffalo; UY: buffaloes from Upper Yangtze region. Details of the sample information are explained in Additional file 1: Table S1

A total of 15 FZ buffaloes were selected for genome resequencing, while 12 UY buffaloes genomic DNA sequences were obtained from previously published data [2] (Additional file 1: Table S1). The clean reads were aligned to the reference genome of buffalo (GCA_003121395.1) with an average alignment rate of 99.06%. The genome resequencing achieved an average depth of 10.54×. In total, 18,216,884 autosomal single nucleotide polymorphisms (SNPs) were identified, including 63,557 nonsynonymous and 128,420 synonymous coding variants (Additional file 2: Table S2). Principal components analysis (PCA) revealed the genetic separation between the FZ and UY buffaloes (Fig. 1).

### Detection of selective sweeps in Fuzhong buffalo

**Fig. 2:**
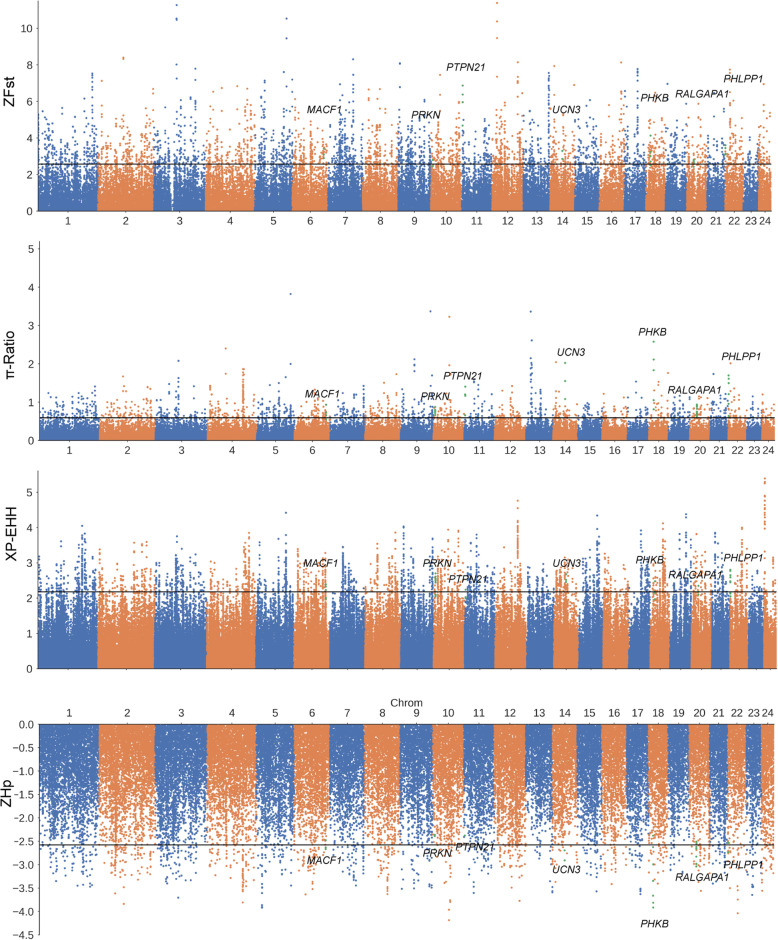
Manhattan plot of selective sweeps in Fuzhong (FZ) buffalo. ZFst, π-Ratio, XP-EHH, and ZHp values were calculated in 50-kb sliding window with 20-kb step across all autosomes in FZ buffalo. The Manhattan plots are pointed the value for each window of ZFst, π-Ratio, XP-EHH and ZHp. The black horizontal line indicates the cut-off (*P*-value < 0.005) setted for extracting outlier for FZ buffalo (ZFst > 2.576; π-Ratio _UY/FZ_ > 0.592, XP-EHH_FZ-to-UY_ > 2.180, and ZH*p <* − 2.576). The green dots represent the regions that contain the highlight gene

**Fig. 3:**
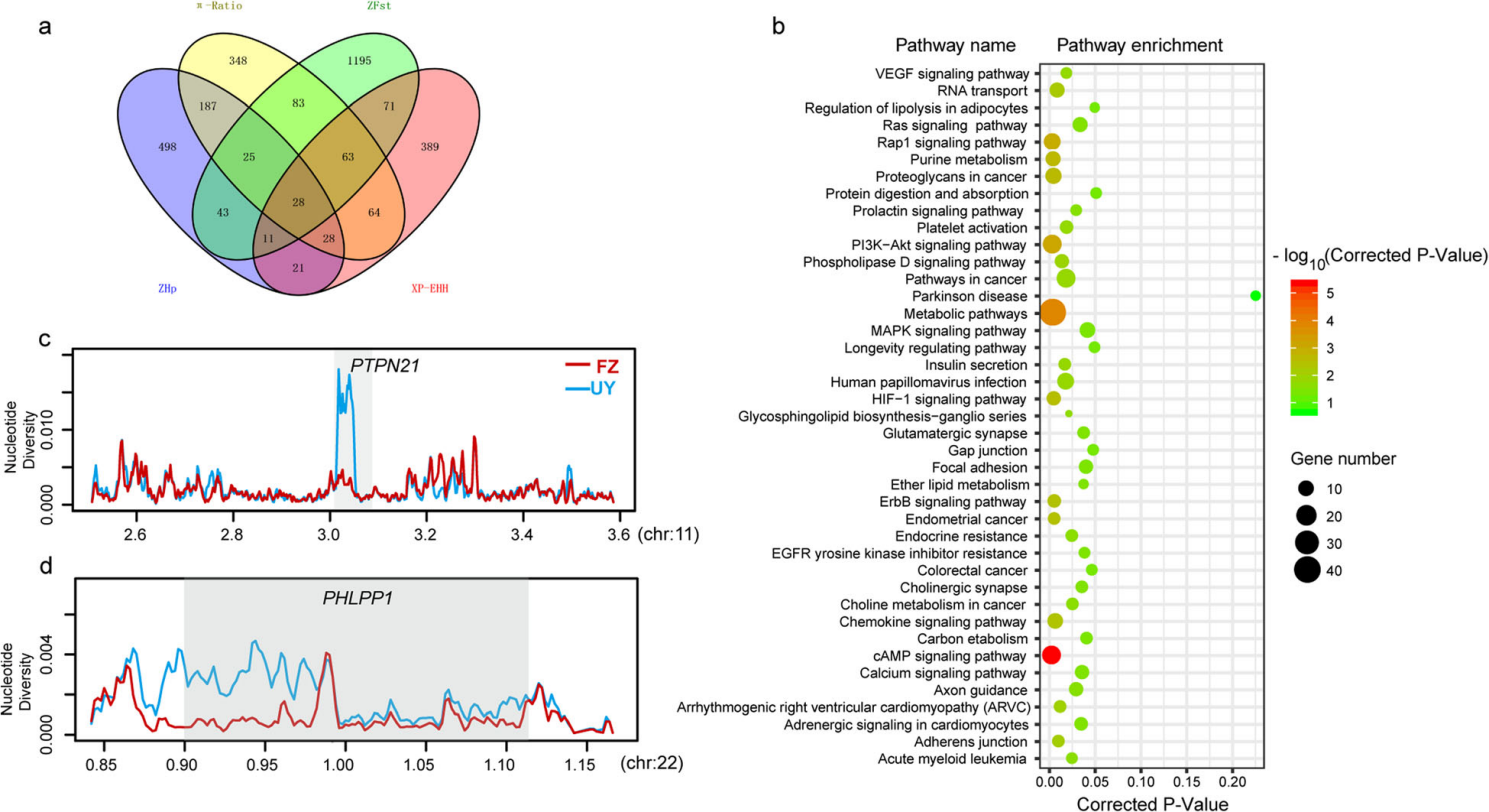
Genomic regions with strong selective signals in FZ buffalo. **a** Number of candidate genes identified in FZ buffalo by the four methods listed in each of the Venn diagram components. **b** KEGG enrichment analysis for the identified candidate genes. Example of genes (**c**, **d**) with selection sweep signals in FZ buffalo. The nucleotide diversity was plotted using a 5-kb sliding window. The gene region was noted by gray shadow in (**c** & **d**)

Due to the genetic separation between FZ and UY buffalo, we performed selective sweep analysis to detect the selection signatures in FZ buffaloes. We used the Fst, Hp, π-Ratio, and XP-EHH tests to search for the genomic regions in FZ buffaloes. Fst is a descriptive statistic and a measure of population genetic differentiation between FZ and UY buffalo. We then calculated the Hp which estimated genetic polymorphism data and was used in population genetic analysis. The Fst and Hp values were normalized (ZFst, ZHp) using the Z-transformation method. Totally, we identified 1519 and 841 candidate genes from ZFst (*P* < 0.005, ZFst > 2.576) and ZHp (*P <* 0.005, ZHp < − 2.576), respectively (Fig. 2, Additional files 3-4: Table S3 and Table S4). In addition, the π-Ratio (*P <* 0.005, π-Ratio > 0.592) and XP-EHH (*P <* 0.005, XP-EHH > 2.180) analysis detected 826 and 675 candidate genes in FZ buffalo, respectively (Additional files 5-6: Table S5 and Table S6). The selective sweeps detected by at least two approaches were defined as the putative selective sweeps. Finally, a total of 599 genes were identified as the candidate genes for FZ buffalo. Among the 599 candidate genes, 21 genes detected by these four different statistics (Fig. 3a). Some of these genes were associated with production and growth traits (*PHLPP1*, *PRKN*, *MACF1*, *UCN3, RALGAPA1*, *PHKB*, *PKD1L2*) (Fig. 2). In addition, the selection signatures in UY buffalo were also detected, resulting in 1519, 949, 1065, and 669 candidate genes from ZFst, ZHp, π-Ratio and XP-EHH analysis, respectively. Totally, 707 overlapping genes were identified as the candidate genes for UY buffalo, while 58 candidate genes were shared between FZ and UY buffalo.

### KEGG pathways and GO enrichments

To obtain a broad overview of the molecular functions of these identified candidate genes for FZ buffalo, KEGG pathways and GO enrichment analysis were performed (Fig. 3b, Additional files 7-8: Table S7 and Table S8). Some of the significant KEGG pathways (corrected *P*-value < 0.05) were associated with the cardiovascular system (arrhythmogenic right ventricular cardiomyopathy (ARVC), corrected *P*-value = 0.0102) and oxidative stress (HIF-1 signaling pathway, corrected *P-*value = 0.0030) (Fig. 3b, Additional files 7-8: Table S7 and Table S8). Several candidate genes (e.g., *ALDOA*, *STAT3*, *AKT2*, *EIF4E2*, *CACNA2D2*, *TCF4*, *CDH2*) were also found to be related to the above pathways.

We also detected significant KEGG pathways (chemokine signaling pathway, corrected *P*-value = 0.0044) and GO terms responsible for immunity (acute myeloid leukemia, corrected *P-*value = 0.0233; positive regulation of defense response to virus by host, corrected *P-*value = 0.0391; leukocyte activation, corrected *P-*value = 0.0442; immune system process, corrected *P-*value = 0.0122) involving immunity related genes (e.g., *NKX2-3*, *PIK3R1*, *ITK*, *TMEM173*, *MTSS1*). In addition, the KEGG pathways (e.g., parkinson disease, corrected *P-*value = 0.2241; glutamatergic synapse, corrected *P-*value = 0.0359; cholinergic synapse, corrected *P-*value = 0.0339) and GO terms (e.g., neuron development, corrected *P-*value = 0.0009; neurogenesis, corrected *P-*value = 0.0079; forebrain neuron differentiation, corrected *P-*value = 0.0455; central nervous system neuron axonogenesis, corrected *P-*value = 0.0454) also enriched genes associated with the nervous system in the FZ buffalo. Several neural-related genes (e.g., *PTPN21*, *ROBO1*, *HOMER1*, *MAGI2*, *SLC1A3*, *NRG3*, *SNAP47*, *CTNNA2*, *ADGRL3*) were also found to be enriched in FZ buffalo.

## Discussion

To date, there have been several studies using the high-throughput sequencing and genotyping technologies to search for candidate genes associated with milk production, growth traits, immune and nervous system of buffaloes [6–9]. However, most of these studies were performed for the dairy river buffalo. The FZ buffalo has been used as a draft animal to provide farm power in rice cultivation. It has also been selected by local people to participate in the bullfight (a folk custom with long history in Guangxi Zhuang Autonomous Region) because of its developed muscles, high endurance, and disease resistance capacity [4]. According to the geographical location, the FZ buffalo belongs to the Southwest China. Our results confirmed the genetic separation between the buffaloes from Southwest China and Upper Yangtze region (Fig. 1) [2].

In this study, we used four methods (ZFst, ZHp, π-Ratio, and XP-EHH) to detect the selective sweeps in FZ buffalo. These four approaches largely belong to two different types. The ZFst, ZHp, π-Ratio were based on allele frequency, while the XP-EHH was based on linkage disequilibrium patterns across genomes. Combining different detection approaches can provide complementary information, which was considered as an optimal strategy in detecting selection signatures [13]. Our results showed that ZHp and π-Ratio detected the most shared genes, while the XP-EHH and ZHp detected the lowest number of shared genes (Fig. 3a). It seems that the number of shared gene between different methods may be not associated with type of method.

FZ buffalo is with strong muscle, able to endure the strength to pull a plough through muddy rice paddies as well as bullfighting. Our results depicted the association of FZ buffalo with oxidative stress, cardiovascular system, and immunity in were several KEGG pathways and GO terms, including HIF-1 signaling pathway, adherens junction, insulin secretion, arrhythmogenic right ventricular cardiomyopathy (ARVC), which were reported to be involved in the reaction to exercise. HIF-1 signaling pathway plays an important role in the regulation of glycolysis during exercise and endurance performance [14]. During endurance training, the endurance exercise can result in oxidative stress and antioxidant defense, and the skeletal muscle experiences severe and repetitive oxygen stress [15]. Studies showed that the metabolic action of insulin was enhanced in skeletal muscle after exercise [16, 17]. Previous reports also indicated that the adherens junction pathway may be related to the exercise in human and horse [18, 19]. ARVC is a rare inherited heart-muscle disease, which could lead to sudden death in young people, athletes, and horse [20, 21]. Previous research has shown the severity of ARVC to be associated with strenuous endurance exercise [22]. In our current study, we identified several candidate genes (e.g., *ALDOA*, *STAT3*, *AKT2*, *EIF4E2*, *CACNA2D2*, *TCF4*, *CDH2*) involved in these two pathways. *ALDOA* (aldolase A, fructose-bisphosphate) is involved in a variety of cellular functions and biological processes, including muscle maintenance, regulation of cell morphology and migration, striated muscle contraction, actin cytoskeleton and actin polymerization and ATP biosynthesis [23–30]. The hypermethylation of *ALDOA* was found to be involved in the anaerobic metabolism in slow muscle fibers [31]. *STAT3* may contribute to the adaptation of skeletal muscle after the acute resistance exercise [32]. *AKT2* is a critical regulator for cardiomyocyte survival and metabolism [33]. *CACNA2D2* plays an important role in heart rate [34], and the *CACNA2D2*-knockout mouse showed lower heart rate [35]. *TCF4* contributes to muscle fiber and basement membrane recovery following the muscle fiber damage induced by exercise [36]. Furthermore, *CDH2* was also identified as a candidate gene involved in cardiomyopathy [37]. All these aforementioned pathways, GO terms, and involved candidate genes directly or indirectly play an important role in oxidative stress and the overall body endurance. These results provided strong evidence that FZ buffalo bear endurance, strong muscular stature and powerful oxidative muscle strength.

Noteworthy KEGG pathway and GO terms (e.g., chemokine signaling pathway, immune system process, leukocyte activation) were over-represented, involving immunity related genes (e.g., *NKX2–3*, *PIK3R1*, *ITK* and *TMEM173*, *MTSS1*). Chemokines are small chemoattractant peptides that provide directional cues for the cell trafficking and thus are vital for protective host response. *PTPN22* plays an important role in autoimmune diseases [38]. *NKX2–3* plays a substantial role in the correct association of lymphocytes and splenic stromal elements [39]. *PIK3R1*, *ITK* and *TMEM173* were identified as potential positional candidate genes associated with infection of *Mycobacterium avium* subspecies paratuberculosis in cattle [40–42]. A study reported that the *MTSS1* was related to metritis in cattle [43]. Guangxi Zhuang Autonomous Region undergoes characteristic subtropical monsoon climate with hot and humid summers and dry and mild winters, which provides an environment for the spread of disease. Although the temperature and humidity are significantly higher than the Yangtze region [3], the native FZ buffalo is with high disease resistance. Our results provided the genetic evidence to illustrate the capability of the local buffalo adaptation to the local environment bearing enhanced disease resistance.

Moreover, the KEGG pathways (e.g., parkinson disease, cholinergic synapse, glutamatergic synapse) and GO terms (e.g., positive regulation of neuron differentiation, forebrain neuron differentiation, central nervous system neuron axonogenesis, regulation of neurogenesis) associated with nerve were detected, involving a set of genes (e.g., *PTPN21*, *ROBO1*, *HOMER1*, *MAGI2*, *SLC1A3*, *NRG3*, *SNAP47*, *CTNNA2*, *ADGRL3*). Moreover, it was found that *PAFAH1B1* was associated with learning, memory, and motor behavior [44]. *ROBO1* was reported to be related to reading disability in humans [45]. A study reported that *ROBO1* was significantly associated with breed-specific accomplishments of dog in competitive obstacle course events [46]. *HOMER1*, *MAGI2*, *SLC1A3*, *NRG3* were associated with schizophrenia [47–50]. *HOMER1* was identified as a candidate gene involving in the domestication of swamp buffalo [2]. *SNAP47* was proved to be involved in postsynaptic and presynaptic function [51]. The *CTNNA2* was associated with excitement-seeking and risk-taking, and were relevant to hyperactivity, substance use, antisocial and bipolar disorders [52]. *ADGRL3* was associated with attention-deficit/hyperactivity disorder [53]. *PTPN21* (protein tyrosine phosphatase, non-receptor type 21), a protein-coding gene which can positively influence cortical neuronal survival and enhance neuritic length [54], was identified as a potential risk gene for schizophrenia [55], showing a reduction in nucleotide diversity in FZ buffalo (Fig. 3c). In addition, we detected a SNP (g. 3,035,884 C > T) in the intron 1 of *PTPN21*. In this locus, the C allele was dominant in FZ buffalo with the frequency of 87.5%, while in buffaloes from Upper Yangtze was rather rare (33.3%). The buffalo is used as a draft animal, which is very gentle and easy to handle and train. In addition, the FZ buffalo was also selected to participate in the traditional folk bullfighting in Guangxi Zhuang Autonomous Region. These results indicated that the nerve of FZ buffalo must have experienced selection, and these genes may act as the selective genes associated with the nerve of FZ buffalo.

Comparing 21 candidate genes detected by 4 methods with the published literature, some of these genes were found to be involved in production and growth traits. *PHLPP1* (PH domain and leucine-rich repeat protein phosphatase 1) was highlighted as functionally plausible candidate gene for pig growth and fatness traits [56, 57], which was also reported to be associated with birth growth trait in cattle [58, 59]. The selective sweep containing *PHLPP1* showed a reduction in nucleotide diversity in FZ buffalo (Fig. 3d). In addition, several genes were associated with meat quality (*PRKN*), feed efficiency (*MACF1*) and carcass traits (*ZNF280B* and *UCN3*) in cattle [60–63]. *RALGAPA1* was relative to reproductive traits in chicken [64]. *PHKB* was as a candidate gene for the feed-conversion ratio in pig [65]. Our results indicated that FZ buffalo experienced artificial breeding, and the growth traits were improved with the artificial selection.

Moreover, 58 candidate genes were found to be shared between FZ buffalo and UY buffalo. In particular, the *TIAM1*, *HOMER1* were identified as the neural-related genes involved in the domestication of swamp buffalo [2]. As we all known, almost all the swamp buffalo is used as the draft animal to provide farm power in rice cultivation, which is very docile and easy to handle and train. Our results further conformed the importance of nervous system for the domestication of swamp buffalo.

## Conclusions

FZ buffalo is a native breed from Guangxi Zhuang Autonomous Region, which is mainly used as a draft animal to plow or level land, puddle rice fields, and bullfight. In this study, we re-sequenced the whole genomes of 15 FZ buffaloes, combining with 12 published buffalo genomes from Upper Yangtze region to detect the selective signatures in FZ buffalo using ZHp, ZFst, π-Ratio, and XP-EHH statistics. Our results depicted several pathways, GO terms, and candidate genes to be associated with response to exercise, immunity, nervous system, and growth traits, which aid us towards a better understanding of the adaptive traits in FZ buffalo. In addition, our results indicated that the nervous system plays an important role in the domestication of swamp buffalo.

## Methods

### Sample collection and sequencing

We sampled a total of 15 ear tissues of FZ buffaloes from Guangxi Zhuang Autonomous Region China. The samples were collected from local farmers, who were interviewed in detail to ensure unrelatedness among the sampled individuals. Following sampling, the animals were returned to their owners. Genomic DNA was extracted from the ear tissue samples using the standard phenol-chloroform protocol. For each sample, 1-15 μg of DNA was used to construct the library with an average insert size of 500 bps. Sequencing was performed to generate 150-bp paired-end reads on an Illumina HiSeq 2000 according to the manufacturer’s protocol. Moreover, 12 publically available buffalo genome sequences [2] from regions of the Upper Yangtze were used as a control group. Detailed information about all samples analyzed in this study was provided in the Additional file 1: Table S1. Raw FASTQ sequences have been deposited to NCBI Short Read Archive under the BioProject accession number PRJNA566371. A study has suggested that around 10 individuals can gain the power for the analysis of selection sweep [66]. Totally, 27 whole genomes of buffalo were used for the further analysis.

### Alignments and variant identification

The clean reads were mapped to the reference genome (GCA_003121395.1) [67] using BWA-MEM with default settings [68]. Furthermore, Samtools, Picard tools, Genome Analysis Toolkit (GATK, version 3.6–0-g89b7209) were used to detect the single nucleotide polymorphisms (SNPs) [69]. All SNPs were filtered using the “Variant Filtration” module of GATK with the standard parameters as below: Variants with quality depth (QD) < 2; FS (Phred-scaled *P*-value using Fisher’s exact test to detect strand bias) > 60; MQRankSum < − 12.5; ReadPosRankSum < − 8; MQ < 40.0; the mean sequencing depth of variants (containing all individuals) < 1/3× and > 3×; SOR > 3.0; maximum missing rate < 0.1; and SNPs were restricted to the two alleles.

### Population structure analyses

The genetic relationships between FZ and UY buffaloes was performed by the principal component analysis (PCA). The SNPs were filtered using the MAF (--maf 0.05) using VCFtools [70]. Moreover, extended parameters (--indep-pairwise 50 5 0.2) of PLINK were used to remove one of the pairs of SNPs if the linkage disequilibrium (LD) with a squared correlation greater than 0.2, in windows of 50 variants and shifting by 5 variants. The SNPs that pass the pruning were used to perform PCA analysis. The PCA was performed using SmartPCA program in the package EIGENSOFT v5.0 [71].

### Genome-wide selective sweep test

To identify the selective sweep regions, the Fst, Hp, π-Ratio, and XP-EHH tests were performed with 50 kb sliding window and 20 kb step. The SNPs were filtered with parameters (--maf 0.05 -max-missing 0.90) using PLINK 1.9 [72]. The Fst [73] was calculated using VCFtools with parameter “--weir-fst-pop group1 --weir-fst-pop group2 --fst-window-size 50000 --fst-window-step 20000 --maf 0.05 --max-missing 0.90” [70], while Hp was calculated as described previously [74]. The Hp and Fst values were converted to a standard normal distribution, denoted by ZHp and ZFst [11]. The genetic diversity (π-Ratio) was calculated using VCFtools with parameters as follows: “--keep gropu1/gropu2 --window-pi 50000 --window-pi-step 20000 --maf 0.05 --max-missing 0.90” [70] and house python scripts. The XP-EHH was performed for every SNP using the default settings by selscan v1.1 [75], and genotypes were phased using Beagle [76] with default parameters. The test statistic was the average normalized XP-EHH score in each 50-kb region. In the π-Ratio, and XP-EHH tests, the FZ buffalo were used as the target population, and the UY buffalo act as the reference population. The *P*-values were estimated based on values of the test using the normal distribution. Significant genomic regions for each method were identified by *P-*value < 0.005. The power of each test was different, any set of candidate genes may contain some false positives [77]. If there is any given signal consistently supported by other methods, it may be considered as a strong evidence that the locus has been under selection. Combining multiple tests can improve the power of detecting selection signatures [78]. To date, almost all the genome-scan-related studies used multiple analyses to further confirm the selected candidate regions to make the results more reliable. In the current study, two or more methods showed outlier signals (*P-*value < 0.005) in overlapping regions and were therefore considered as the candidate selective regions and were subsequently examined for the candidate genes [79]. The candidate regions were separated by a distance with less than 50 kb using the house script. In order to gain better understanding of the biological functions and pathway of the identified candidate genes, the Kyoto Encyclopedia of Genes and Genomes (KEGG) pathway and Gene Ontology (GO) were performed using KOBAS 3.0 [80], considering a significant threshold for corrected *P-*value < 0.05.

## Supplementary information

**Additional file 1: Table S1.** Overview of sample information and sequencing statistics.

**Additional file 2: Table S2**. Distribution of SNPs within various genomic regions.

**Additional file 3: Table S3**. A summary of genes from ZFst.

**Additional file 4: Table S4**. A summary of genes from ZHp.

**Additional file 5: Table S5**. A summary of genes from π-Ratio.

**Additional file 6: Table S6**. A summary of genes from XP-EHH.

**Additional file 7: Table S7**. KEGG pathway analysis of candidate genes in FZ buffalo.

**Additional file 8: Table S8**. GO enrichment analysis of candidate genes in FZ buffalo.

## Data Availability

Raw FASTQ sequences have been deposited to NCBI Short Read Archive under the BioProject accession number PRJNA566371.

## Abbreviations

FZ: Fuzhong
UY: Upper Yangtze
KEGG: Kyoto Encyclopedia of Genes and Genomes
GO: Gene Ontology
Fst: The genetic differentiation
Hp: Heterozygosity
π-Ratio: Genetic diversity
XP-EHH: Cross-population extended haplotype homozygosity
PCA: Principal component analysis
